# Visceral Leishmaniasis Relapse in Southern Sudan (1999–2007): A Retrospective Study of Risk Factors and Trends

**DOI:** 10.1371/journal.pntd.0000705

**Published:** 2010-06-08

**Authors:** Stanislaw Gorski, Simon M. Collin, Koert Ritmeijer, Kees Keus, Francis Gatluak, Marius Mueller, Robert N. Davidson

**Affiliations:** 1 Médecins Sans Frontières, Amsterdam, The Netherlands; 2 Department of Social Medicine, University of Bristol, Bristol, United Kingdom; 3 Department of Infection and Tropical Medicine, Northwick Park Hospital, Harrow, United Kingdom; Institute of Tropical Medicine, Belgium

## Abstract

**Background:**

Risk factors associated with *L. donovani* visceral leishmaniasis (VL; kala azar) relapse are poorly characterized.

**Methods:**

We investigated patient characteristics and drug regimens associated with VL relapse using data from Médecins Sans Frontières - Holland (MSF) treatment centres in Southern Sudan. We used MSF operational data to investigate trends in VL relapse and associated risk factors.

**Results:**

We obtained data for 8,800 primary VL and 621 relapse VL patients treated between 1999 and 2007. Records of previous treatment for 166 VL relapse patients (26.7%) were compared with 7,924 primary VL patients who had no record of subsequent relapse. Primary VL patients who relapsed had larger spleens on admission (Hackett grade ≥3 vs0, odds ratio (OR) for relapse = 3.62 (95% CI 1.08, 12.12)) and on discharge (Hackett grade ≥3 vs 0, OR = 5.50 (1.84, 16.49)). Age, sex, malnutrition, mobility, and complications of treatment were not associated with risk of relapse, nor was there any trend over time. Treatment with 17-day sodium stibogluconate/paromomycin (SSG/PM) combination therapy vs 30-day SSG monotherapy was associated with increased risk of relapse (OR = 2.08 (1.21, 3.58)) but reduced risk of death (OR = 0.27 (0.20, 0.37)), although these estimates are likely to be residually confounded. MSF operational data showed a crude upward trend in the proportion of VL relapse patients (annual percentage change (APC) = 11.4% (−3.4%, 28.5%)) and a downward trend in deaths (APC = −18.1% (−22.5%, −13.4%)).

**Conclusions:**

Splenomegaly and 17-day SSG/PM vs 30-day SSG were associated with increased risk of VL relapse. The crude upward trend in VL relapses in Southern Sudan may be attributable to improved access to treatment and reduced mortality due to SSG/PM combination therapy.

## Author Summary

Visceral leishmaniasis (kala-azar) caused by *Leishmania donovani* is spread from person to person by *Phlebotomus* sandflies. Major epidemics of visceral leishmaniasis have occurred in Southern Sudan during the 20th century. The worst of these killed 100,000 people in the western Upper Nile area of Southern Sudan from 1984–1994, a loss of one-third of the population. Médecins Sans Frontières has treated 40,000 kala-azar patients in Southern Sudan since the late 1980's. In this study we used routinely collected clinical data to investigate why some patients relapse after treatment. We found that patients with severely enlarged spleens (splenomegaly) are more likely to relapse. Patients treated for 17 days with a combination of two drugs (sodium stibogluconate and paromomycin) were more likely to relapse (but less likely to die) than patients treated for 30 days with a single drug (sodium stibogluconate). However, the transition from sodium stibogluconate to the sodium stibogluconate/paromomycin combination as standard treatment between 2001–2003 has not led to a significant increase in visceral leishmaniasis relapse.

## Introduction

Visceral leishmaniasis (VL, kala-azar) is a systemic parasitic disease caused by the *Leishmania donovani* species complex. VL manifests with irregular bouts of fever, substantial weight loss, hepatosplenomegaly, pancytopenia, and susceptibility to opportunistic infection [1]. VL is typically fatal unless treated. In immunocompetent individuals, effective drug treatment reduces *Leishmania* amastigotes to a level undetectable in aspirates. An effective life-long cellular immune response normally develops, and residual parasites are suppressed [2]. Despite apparent clinical and parasitologic response to treatment, a proportion of VL patients who are apparently otherwise immunocompetent, have a recurrence of VL. This usually occurs within 6 months of treatment [1], [3], and later recurrence is rare – suggesting that recrudescence rather than re-infection is the usual mechanism of relapse. While the role of HIV infection in VL relapse is well-documented, for example among patients in southern Europe, risk factors for VL relapse among HIV-negative patients in VL-endemic regions of Africa remain poorly characterised.

Médecins Sans Frontières - Holland (MSF) has treated >80,000 VL patients in Sudan and Ethiopia since 1989, and has maintained an electronic record of patient characteristics, treatments and outcomes. Although these routinely-collected data are intrinsically incomplete due to the very challenging environment in which they are collected, our previous analyses have yielded important findings [3]–[10]. Most recently, we analysed risk factors for VL relapse in patients in Northern Ethiopia who were co-infected with HIV [11]. In contrast, the prevalence of HIV in Southern Sudan has remained the lowest in Africa until very recently [12]. Here we investigate risk factors for VL relapse among patients from this largely HIV-negative population who were treated at MSF clinics in Southern Sudan from 1999 to 2007.

## Methods

### Diagnosis

Diagnostic, treatment, and discharge procedures used were consistent with WHO guidelines [13]. VL was diagnosed in clinical suspects by high titer (≥1∶6,400) antibodies to *Leishmania* (freeze-dried *Leishmania* antigen supplied by the Royal Tropical Institute, Amsterdam, The Netherlands) in a direct agglutination test (DAT); or by microscopy of splenic or lymph node aspirates; or (since 2004) by rK39 rapid diagnostic test (DiaMed-IT-Leish supplied by DiaMed AG, Cressier sur Morat, Switzerland); or on rare occasions when laboratories were not functioning, by clinical judgment (criteria: fever >2 weeks with exclusion of malaria and either splenomegaly or lymphadenopathy and wasting).

### Treatment

Until 2002, standard treatment for primary VL comprised daily IM injections of sodium stibogluconate (SSG; Albert David Ltd, Calcutta; supplied by the International Dispensary Association, Amsterdam, The Netherlands) at a dose of 20mg/kg/day (minimum dose, 200mg; no maximum dose) for 30 days. Between 2001 and 2003, SSG monotherapy as standard treatment was replaced with combination therapy of paromomycin (PM) plus SSG. This comprised 17 daily intramuscular injections of SSG 20mg/kg and paromomycin sulfate (Pharmamed Parenterals Ltd, Malta, supplied by the International Dispensary Association, Amsterdam, The Netherlands) at a dose of 15mg/kg (equivalent to ∼11mg/kg of PM base). SSG/PM was initially withheld from females of childbearing age, and since 2004 withheld only from pregnant women. SSG/PM was administered only at MSF treatment centres with a permanent expatriate doctor, and these centres also had access to parenteral antibiotics and therapeutic feeding. In 2003 MSF introduced a formal risk assessment using a scoring system based on known risk factors for adverse outcomes [3]. Thereafter, critically ill patients at high risk of death were treated initially in special care areas with rehydration, therapeutic nutrition, antibiotics, and liposomal amphotericin B (AmBisome®, Gilead Pharmaceuticals); when their condition improved, treatment continued with SSG/PM. Before the introduction of this “combined AmBisome-paromomycin-stibogluconate treatment” (CAPST) in 2003, treatment with AmBisome was unusual. The prohibitive cost of AmBisome was reduced for public sector agencies in developing countries in 2007, allowing all critically ill patients to be treated with a full course of AmBisome.

### Discharge

Cases of primary VL who did not respond clinically to treatment underwent a test-of-cure aspirate from spleen or lymph node. If the test-of-cure result was positive, daily SSG injections were continued until two consecutive weekly test-of-cure results were negative. Patients whose aspirate remained positive after ≥60 SSG injections were treated with combinations of SSG, PM and AmBisome. On discharge, patients were given an identification card to be presented in case of re-admission for relapse or for post kala-azar dermal leishmaniasis (PKDL).

### Re-admission

Patients with possible relapse of VL (i.e. patients who presented their identification card from previous treatment or who verbally reported previous treatment) were diagnosed by splenic or lymph node aspirate. Relapsed VL patients were treated with 17 days of SSG/PM plus further daily injections of SSG until two weekly test-of-cure results were negative. Patients who relapsed ≥2 times were treated with 6 IV doses of 4–6mg/kg of AmBisome on alternate days. In patients with relapses, tuberculosis (TB) and HIV were considered as possible co-factors and appropriate tests done. In rare cases of repeated VL relapses, empiric TB treatment was sometimes given. Patients with severe PKDL were treated with SSG or, if not responding, a combination of SSG/PM, until their condition improved.

### Data sources

Since 1999 we have made an electronic archive of VL patient records from MSF treatment centres in Southern Sudan. Demographic, diagnostic, treatment, and discharge data were handwritten on a card during a patient's stay at a treatment centre. MSF staff at our Lokichoggio base on the Sudan/Kenya border entered data from these cards into EpiInfo, version 6 (Centres for Disease Control and Prevention). Due to time and resource constraints, all data were single-entered. During the period 1999–2007, MSF treatment centres were situated in Upper Nile State (Abuong, Atar, Bimbim, Magang, Rupbuot, Wudier), Jonglei State (Lankien, Pieri, Pading), and Unity State (Bil, Leer, Nimne, Thonyor). For analysis of trends we used MSF operational data, which provided monthly summary statistics on total VL treatment activity (admissions, diagnoses and outcomes) at each treatment centre.

### Data cleaning

Anomalous, inconsistent, or missing values were identified and corrected if possible, otherwise recoded as missing. Adult (age >19 years) body mass index (BMI) values were re-calculated from weight and height data. Weight-for-height (WFH) Z-scores for children age <5 years were generated using WHO Anthro 2005 software (World Health Organisation, Geneva). BMI-for-age Z-scores for patients 5–19 years old were calculated using WHO Survey 2007 software (World Health Organisation, Geneva) [14].

### Inclusion criteria

This study was concerned only with patients with primary VL (i.e. no record of previous treatment) during 1999–2007. In the first year (1999), Lankien was the only functioning VL treatment centre in the area, with only 443 primary VL admissions and a recorded relapse rate of 3.7% by passive case-finding; we consider it unlikely that any of these primary VL patients were misdiagnosed VL relapse patients who had been treated elsewhere, because VL treatment had not been available elsewhere in the region. Patients who subsequently relapsed were identified by matching their primary and relapse VL treatment records, using their patient identifier, sex and age. Patients who died or defaulted (self-discharge against medical advice) during treatment were excluded from our analysis of risk factors for relapse.

### Outcomes

Our primary outcome was relapse after treatment for primary VL. We also analysed death during VL treatment as a secondary outcome in a comparison of SSG/PM *vs* SSG.

### Variables

We used the following admission data: age, sex, treatment centre, calendar year, self-reported duration of illness (‘time-to-presentation’), walking status for patients ≥5 years old (walking normally, with a stick, or with assistance or carried on stretcher - a marker for general weakness). Data on admission and discharge included: body mass index (BMI) (for patients >19 years old), BMI-for-age Z-score (for patients 6–19 years old) [14], weight-for-height (WFH) Z-score (for patients ≤5 years old), spleen size (Hackett grade) [15], and haemoglobin (g/dl) (measured by color densitometry before 2006; Haemocue thereafter). Malnutrition in appropriate age groups was defined as: BMI<14kg/m^2^; BMI-for-age Z-score<−3; and WFH Z-score<−3. Treatment data included: drug used and occurrence of diarrhoea, bleeding and vomiting. The majority of patients were treated at one centre (Lankien) which differed from the other (smaller) treatment centres in that expatriate and/or experienced national medical staff were stationed there permanently (except when evacuated for reasons of security). Hence, treatment centre was coded dichotomously to indicate Lankien or ‘other’ treatment centre.

### Statistical analysis

We compared patients and outcomes by Chi-squared test and t-test. Odds ratios for VL relapse, adjusted a-priori for age, sex, treatment centre, and time (calendar year), were estimated for all variables; those variables which were associated with VL relapse were then included, with the a-priori confounders, in a multivariable logistic regression model. All statistical analyses were performed using Stata Release 10 (StataCorp. 2007. *Stata Statistical Software: Release 10*. College Station, TX). Trends based on MSF operational data (summary statistics) were analysed using US National Cancer Institute Join-point regression software, version 3.0 (http://srab.cancer.gov/joinpoint/), which performs linear regression to estimate the annual percent change (APC) in the dependent variable (rates are assumed to change at a constant percentage of the rate of the previous year, ie. to change linearly on a log scale) and the number and location of join-points (points at which trends change) [16]. The software performs pair-wise comparisons of models differing by one join-point to determine the model with the optimum fit to the data series. An overall significance level of 5% was adopted for the comparisons of models applied to each data series. We tested different permutations of the maximum number of join-points (2, 3 or 4), the minimum number of observations between joinpoints (3 or 4) and the minimum number of observations between joinpoints and the ends of the data (2 or 3).

### Sensitivity analyses

(1) Patients without an enlarged spleen could not undergo spleen aspirate, and lymph node aspirates were performed by MSF at only one centre (Lankien), hence we suspected that some VL patients diagnosed by DAT alone may have been misdiagnosed, because DAT has a false positive rate of ≥10% [17], [18]. We therefore performed a sensitivity analyses, excluding patients without splenomegaly on admission, to assess the degree of mis-diagnosis. (2) The transition from SSG to SSG/PM in the second half of the period in our study was accompanied by an increase in the proportion of VL relapse patients for whom a record of previous treatment could be identified. In order to assess possible bias in estimating the association of SSG/PM *vs* SSG with risk of relapse, we performed a sensitivity analysis in which we assumed that VL relapse patients for whom a record of previous treatment could not be identified (hence, who were excluded from the main analysis of risk factors for relapse) had the same characteristics on admission for primary VL (same age, sex, treatment centre, time of treatment and treatment with SSG or SSG/PM) as they did on admission for relapse VL.

### Ethics approval

Data were collected as part of routine patient care; no additional investigations were performed. Ethical approval was obtained from the MSF Ethical Review Board.

## Results

### Characteristics of patients

**Figure 1:**
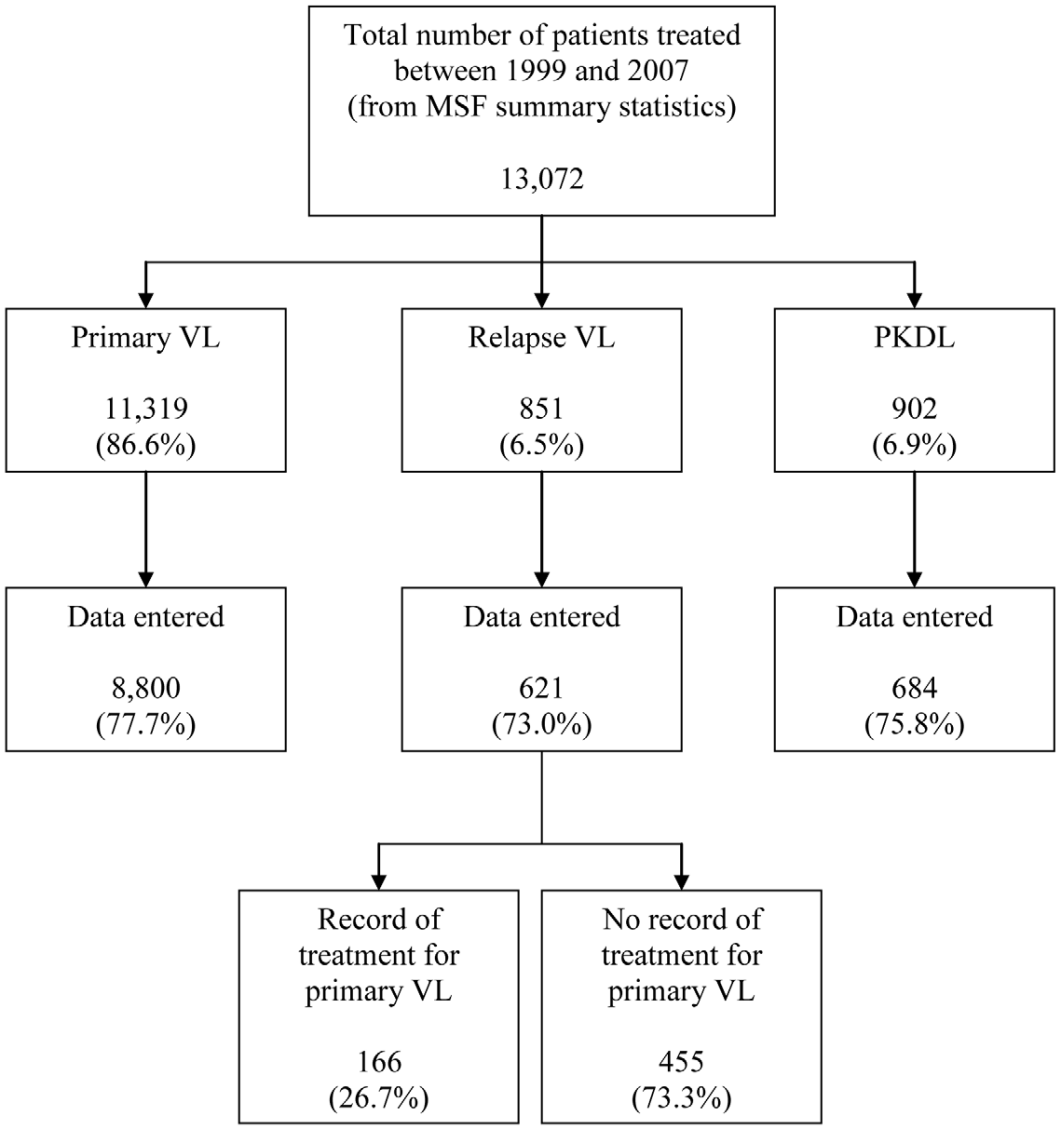
Patients treated by MSF in Southern Sudan (1999–2007) who were included in this study.

The treatments for which electronic archive data were available are summarised in **Table 1**. Overall, records were available for 78.2% (10,105/12,924) of treatments, according to MSF monthly field activity reports. For relapse VL, the corresponding proportion of treatments with entered data was higher, at 88.1% (621/705) (**Figure 1**). The Lankien and Pieri treatment centres were looted in 2004 and 2006, respectively, with the loss or destruction of many treatment cards. Other losses of treatment cards were due to generally adverse conditions in the field and difficulties in collecting and transporting paper records from remote sites, some of which had no permanent expatriate presence.

**Table 1:** Primary VL, relapse VL and PKDL treatment data from Médecins Sans Frontières - Holland clinics in Southern Sudan (1999–2007).

	1999	2000	2001	2002	2003	2004	2005	2006	2007	Total
**Primary VL**	636	346	1,514	1,766	2,620	1,057	456	354	51	8,800
	(86.0%)	(77.1%)	(89.4%)	(86.2%)	(90.8%)	(84.6%)	(88.2%)	(77.5%)	(78.5%)	(87.1%)
**Relapse VL**	31	54	53	132	165	79	25	72	10	621
	(4.2%)	(12.0%)	(3.2%)	(6.4%)	(5.8%)	(6.3%)	(4.8%)	(15.8%)	(15.4%)	(6.1%)
**PKDL**	73	49	126	151	101	113	36	31	4	684
	(9.9%)	(10.9%)	(7.5%)	(7.4%)	(3.5%)	(9.0%)	(7.0%)	(6.8%)	(6.1%)	(6.8%)
**Total for whom treatment data were entered**	740	449	1,693	2,049	2,886	1,249	517	457	65	10,105
	(100.0%)	(100.0%)	(100.0%)	(100.0%)	(100.0%)	(100.0%)	(100.0%)	(100.0%)	(100.0%)	(100.0%)
**Total number of treatments** a	799	540	1,909	2,111	3,414	1,934	1,447	590	180	12,924
**Proportion for whom data were entered**	92.6%	83.1%	88.7%	97.1%	84.5%	64.6%	35.7%	77.5%	36.1%	78.2%
**Proportion of primary VL patients treated in Lankien** b	58.4%	45.4%	41.2%	80.9%	80.4%	4.8%	75.0%	76.8%	98.0%	61.4%

a from MSF summary statistics.

b low proportion in 2004 due to looting of Lankien and consequent loss of patient medical record.

Our analysis was based on primary VL patients who were known to have subsequently relapsed because a record of treatment for VL relapse could be linked with their primary VL treatment record (**Figure 1**). These relapse patients (N = 166) were a subset (26.7%) of all VL relapse patients who had an electronic record of treatment for relapse VL (N = 621). **Table 2** shows a comparison of the characteristics of VL relapse patients who did or did not have a record of previous treatment for primary VL. Patients for whom we could not identify a previous treatment record had more missing data, higher adult BMI, longer duration of illness, and less splenomegaly than patients whose previous treatment data were used in our analysis of factors associated with VL relapse, but there were no differences in age, sex, nutritional status of patients ≤19 years old, haemoglobin, walking status, drug treatment, or treatment outcome. More than half (57.8%) of the 621 VL relapse patients were treated at one centre (Lankien), and a record of previous treatment was available for 34.3% (123/359) of these patients compared with 16.4% (43/262) for all other treatment centres combined (P<0.001). A record of previous treatment was more likely to be identified in the second half of the period under study (36.5% in 2003–2007) compared with the first half (14.1% in 1999–2002) (P<0.001).

**Table 2:** Characteristics of relapse VL patients (N = 621) who did or did not have a record of previous treatment for primary VL.

		Relapse VL patients who had a record of treatment for primary VL (N = 166)	Relapse VL patients who did not have a record of treatment for primary VL (N = 455)	P-value*
Age group	<5 years	18.7%	20.9%	0.8
	5–19 years	47.6%	46.4%	
	>19 years	33.7%	32.8%	
Sex	Male	59.6%	57.1%	0.6
Treatment centre	Lankien	74.1%	51.9%	<0.001
	Other	25.9%	48.1%	
Mean BMI (patients >19 years) (kg/m^2^)		(n = 51) 15.24	(n = 108) 16.14	0.01
Mean BMI-for-age Z-score (patients 5–19 years)		(n = 65) −3.34	(n = 149) −3.05	0.3
Mean WFH Z-score (patients <5 years)		(n = 31) −2.21	(n = 86) −2.53	0.4
Malnourished (Z-score<−3 or BMI<14kg/m^2^)		41.6%	34.9%	0.2
*Missing height or weight data*		*11.5%*	*24.6%*	*<0.001*
Mean hemoglobin (g/dl)		8.32	8.27	0.9
*Missing hemoglobin data*		*54.2%*	*73.9%*	*<0.001*
Mean duration of illness (months)		1.8	2.5	<0.001
*Missing duration of illness data*		*5.4%*	*5.1%*	*0.9*
Spleen size (Hackett Grade)	0	9.6%	11.2%	0.003
	1	7.2%	19.1%	
	2	35.5%	34.7%	
	≥3	42.2%	30.6%	
	*Missing*	*5.4%*	*4.4%*	
Walking status	Unassisted	31.9%	34.5%	1.0
	With a stick	40.4%	38.7%	
	Assisted/Stretcher	10.2%	9.9%	
	*Missing*	*17.5%*	*16.9%*	
Drug used for VL relapse	SSG	19.3%	28.6%	0.1
	SSG/PM	72.3%	65.5%	
	Other/CAPST	7.2%	5.1%	
	*Missing*	0.9%	1.2%	
Treatment outcome	Discharged	88.6%	87.7%	0.7
	Died	6.0%	5.3%	
	Defaulted	5.4%	7.0%	

*Student's t-test for difference between mean values, Chi-squared test for differences in proportions.

### Factors associated with VL relapse among patients treated for primary VL

**Table 3** shows a comparison of the characteristics on admission of primary VL patients who did or did not have a recorded subsequent relapse. Higher odds of relapse were associated with splenomegaly (on admission for, and discharge from, treatment for primary VL) and with SSG/PM treatment of primary VL. In a logistic regression model which included age, sex, year and treatment centre, admission spleen size (Hackett grade) ≥3 was associated with >4-fold higher odds of relapse compared with size 0 (odds ratio (OR) = 4.40 (95% CI 1.74–11.08), P = 0.002). Admission spleen grades 1 and 2 were associated with >2-fold and 3-fold higher odds: OR = 2.43 (0.95–6.26), P = 0.02 for size 1 *vs* size 0; OR = 2.91 (1.17–7.24), P = 0.07 for size 2 *vs* size 0. In a similarly-adjusted model, 17-day SSG/PM was associated with >2-fold higher odds of relapse (OR = 2.26 (1.46–3.51), P<0.001) compared with 30-day SSG monotherapy. This estimate was robust to a sensitivity analysis in which the N = 455 VL relapse patients for whom no record of previous treatment was available were included in the dataset as primary VL patients who subsequently relapsed, under the assumption that these patients were treated with the same drug for both VL episodes (OR = 2.25 (1.79–2.82), P<0.001).

**Table 3:** Characteristics on admission of primary VL patients who did or did not have record of subsequent relapse.

		Primary VL patients who subsequently relapsed (N = 166)	Primary VL patients who had no record of subsequent relapse (N = 7,924)	P-value*
Age group	<5 years	21.1%	17.7%	0.5
	5–19 years	45.8%	46.3%	
	>19 years	33.1%	36.0%	
Sex	Male	58.4%	52.9%	0.2
Treatment centre	Lankien	77.7%	60.4%	<0.001
	Other	22.3%	39.6%	
Mean BMI (patients >19 years) (kg/m^2^)		(n = 54) 15.50	(n = 2,525) 15.57	0.8
Mean BMI-for-age Z-score (patients 5–19 years)		(n = 65) −3.48	(n = 2,882) −3.40	0.6
Mean WFH Z-score (patients <5 years)		(n = 30) −2.65	(n = 1,254) −2.68	0.9
Malnourished (Z-score<−3 or BMI<14kg/m^2^)		26.8%	28.6%	0.6
*Missing height or weight data*		*10.2%*	*15.9%*	*0.01*
Mean admission hemoglobin (g/dl)		8.26	8.66	0.1
*Missing hemoglobin data*		*67.5%*	*68.4%*	*0.8*
Mean duration of illness (months)		1.73	1.88	0.2
*Missing duration of illness data*		*1.8%*	*3.1%*	*0.4*
Admission spleen size (Hackett Grade)	0	3.0%	8.1%	0.002
	1	20.5%	25.0%	
	2	44.0%	42.8%	
	≥3	31.3%	20.8%	
	*Missing*	*1.2%*	*3.4%*	
Walking status	Unassisted	22.9%	23.1%	1.0
	With a stick	48.2%	49.2%	
	Assisted/Stretcher	15.7%	14.8%	
	*Missing*	*13.3%*	*13.0%*	
Drug used for primary VL	SSG	27.1%	43.9%	<0.001
	SSG/PM	70.5%	54.8%	
	Other/CAPST	0.0%	0.5%	
	*Missing*	*2.4%*	*0.8%*	
Vomiting during treatment		28.3%	28.2%	1.0
Bleeding during treatment		2.4%	3.8%	0.4
Diarrhoea during treatment		34.3%	35.2%	0.8

*Student's t-test for difference between mean values, Chi-squared test for differences in proportions.

Young age (<5 years) was not associated with risk of relapse. However, among children <5 years old, infancy (age <1 year) compared with age 1–4 years was associated with higher risk of relapse (OR = 3.47 (1.16–10.37), P = 0.03). Patients treated at Lankien were more than twice as likely to relapse (OR = 2.32 (1.61–3.36), P<0.001) as patients treated at any other centre (model adjusted for age, sex and year of treatment).

Test-of-cure data were available for only 4.2% (7/166) of relapse patients who had a previous treatment record *vs* 5.4% (431/7,924) of patients with no record of subsequent relapse (P = 0.6). None of the final test-of-cure results for relapse patients, and only 3/431 of the test-of-cure results for patients with no record of subsequent relapse were positive. Hence, test-of-cure data were not included in the risk factor analysis. Whether or not a test-of-cure was performed (indicative of non-response to treatment) was not associated with risk of relapse (OR = 1.19, 95% CI 0.60–2.34).

Patients who did not have a recorded relapse were missing more height and weight data, but none of the anthropometric measures on admission were associated with VL relapse. Although mean hemoglobin was not associated with VL relapse, in multivariable regression (adjusted for age, sex, year and treatment centre) primary VL patients presenting with hemoglobin in the lowest quartile (≤7.0g/dl) had an odds ratio for relapse of 2.60 (1.00–6.74), P = 0.05) compared with patients in the highest quartile (>10g/dl). Splenomegaly and anemia were strongly correlated (decrease in Hb concentration for each increment in Hackett grade = 0.47 (0.40–0.54) g/dl, P<0.001, by linear regression adjusted for age and sex).

Higher risk of relapse was also strongly associated with spleen size at end of treatment (OR = 5.41 (1.89–15.47), P = 0.002 for size ≥3 *vs* size 0; OR = 2.09 (1.19–3.67), P = 0.01 for size 2 *vs* size 0; OR = 1.45 (0.82–2.53), P = 0.2 for size 1 *vs* size 0) (**Table 4**). These associations were slightly attenuated if we excluded patients who had no palpable spleen or no spleen size data on admission (OR = 5.01 (1.75–14.37), P = 0.003 for size ≥3 *vs* size 0; OR = 1.98 (1.12–3.48), P = 0.02 for size 2 *vs* size 0; OR = 1.31 (0.74–2.34), P = 0.4 for size 1 *vs* size 0). Spleen size at end of treatment for primary VL was not correlated with spleen size on admission for relapse VL (pairwise correlation coefficient = 0.12, P = 0.2).

**Table 4:** Characteristics on discharge of primary VL patients who did or did not have record of subsequent relapse.

		Primary VL patients who subsequently relapsed (N = 166)	Primary VL patients who had no record of subsequent relapse (N = 7,924)	P-value*
Mean BMI (patients >19 years) (kg/m^2^)		(n = 30) 15.88	(n = 1,465) 16.14	0.8
Mean BMI gain(+)/loss (−) during treatment (kg/m^2^)		+0.43 (P = 0.2)	+0.33 (P<0.001)	
Mean BMI-for-age Z-score (patients 5–19 years)		(n = 33) −2.93	(n = 1,769) −2.96	0.9
Mean BMI-for-age Z-score gain(+)/loss (−) during treatment		+0.36 (P = 0.01)	+0.49 (P<0.001)	
Mean WFH Z-score (patients <5 years)		(n = 17) −2.03	(n = 725) −2.17	0.7
Mean WFH Z-score gain(+)/loss (−) during treatment		+0.83 (P = 0.1)	+0.57 (P<0.001)	
Malnourished (Z-score<−3 or BMI<14kg/m^2^)		42.0%	42.0%	1.0
Mean percentage gain in body weight (%) - all ages		2.6%	4.3%	0.07
*Missing body weight gain data*		*51.8%*	*50.0%*	*0.05*
Mean percentage gain in hemoglobin (%)		18.0%	18.2%	1.0
*Missing hemoglobin gain data*		*79.8%*	*77.5%*	*0.5*
Discharge spleen size (Hackett Grade)	0	49.4%	58.3%	0.005
	1	9.0%	8.0%	
	2	9.0%	5.6%	
	≥3	2.4%	0.6%	
	*Missing*	*30.1%*	*27.4%*	

*Student's t-test for difference between mean values, Chi-squared test for differences in proportions.

There were gains in BMI and WFH during treatment among patients who did and who did not have a subsequently recorded relapse, although these gains were statistically evident (P<0.001) only for the (much larger) group of non-relapsing patients. Weight on discharge was recorded for only 50% of patients. Among patients of all ages, the mean gain in body weight (as a percentage of weight on admission) was higher in non-relapsing patients (4.2%) than in relapsing patients (2.4%). In a linear regression model (adjusted for age, sex, year, treatment centre, and weight on admission), the difference in percentage weight gain between non-relapsing and relapsing patients was 2.29% (95% CI 0.43%–4.14%; P = 0.02).

In a multivariable model (**Table 5**) comprising age, sex, year, treatment centre, spleen size on admission, spleen size on discharge, and treatment given (SSG/PM *vs* SSG): splenomegaly Hackett grade ≥3 on admission for, and on discharge from, treatment for primary VL were independently associated with higher odds of subsequent relapse compared with size 0 (on admission OR = 3.62 (1.08–12.12), P = 0.04; on discharge OR = 5.50 (1.84–16.49), P = 0.002); SSG/PM was associated with higher odds of relapse (OR = 2.08 (1.21–3.58), P = 0.008); and patients treated at Lankien were more likely to relapse (OR = 1.76 (1.14–2.72), P = 0.01) than patients treated at any other centre. In a sensitivity analysis, excluding patients who had spleen size 0 or missing spleen size data on admission from this model (**Table 5**), the association of splenomegaly on discharge with higher odds of relapse was slightly attenuated (OR = 5.38 (1.79–16.12), P = 0.003 for size ≥3 *vs* size 0), as was the association of SSG/PM with higher odds of relapse (OR = 2.02 (1.16–3.51), P = 0.01). SSG/PM compared with SSG monotherapy was associated with 73% lower odds of death among primary VL patients (OR = 0.27 (0.20–0.37), P<0.001) and 85% lower odds of death among relapse VL patients (OR = 0.15 (0.05–0.46), P = 0.001), in models adjusted for age, sex, year and treatment centre.

**Table 5:** Multivariable model of risk factors for relapse after treatment for primary VL.

		Odds ratio*	Odds ratio in sensitivity analysis**
		(95% CI)	(95% CI)
Admission spleen size (Hackett Grade)	0	1.00	-
	1	3.48 (1.05–11.53)	1.00
	2	3.27 (1.01–10.55)	0.95 (0.54–1.66)
	≥3	3.62 (1.08–12.12)	1.05 (0.60–1.84)
	Test-for-trend***	P = 0.1	P = 0.9
Discharge spleen size (Hackett Grade)	0	1.00	1.00
	1	1.47 (0.83–2.60)	1.37 (0.76–2.47)
	2	1.88 (1.02–3.49)	1.88 (1.02–3.48)
	≥3	5.50 (1.84–16.49)	5.38 (1.79–16.12)
	Test-for-trend***	P = 0.003	P = 0.002
Use of SSG/PM *vs* SSG for primary VL		2.08 (1.21–3.58)	2.02 (1.16–3.51)

*adjusted for age, sex, year, treatment centre and all variables in table.

**excluding patients who had spleen size ‘0’ on admission (adjusted for age, sex and all variables in table).

***Wald test-for-trend across spleen size as linear variable.

### Trends in VL relapse and related factors in Southern Sudan

**Figure 2:**
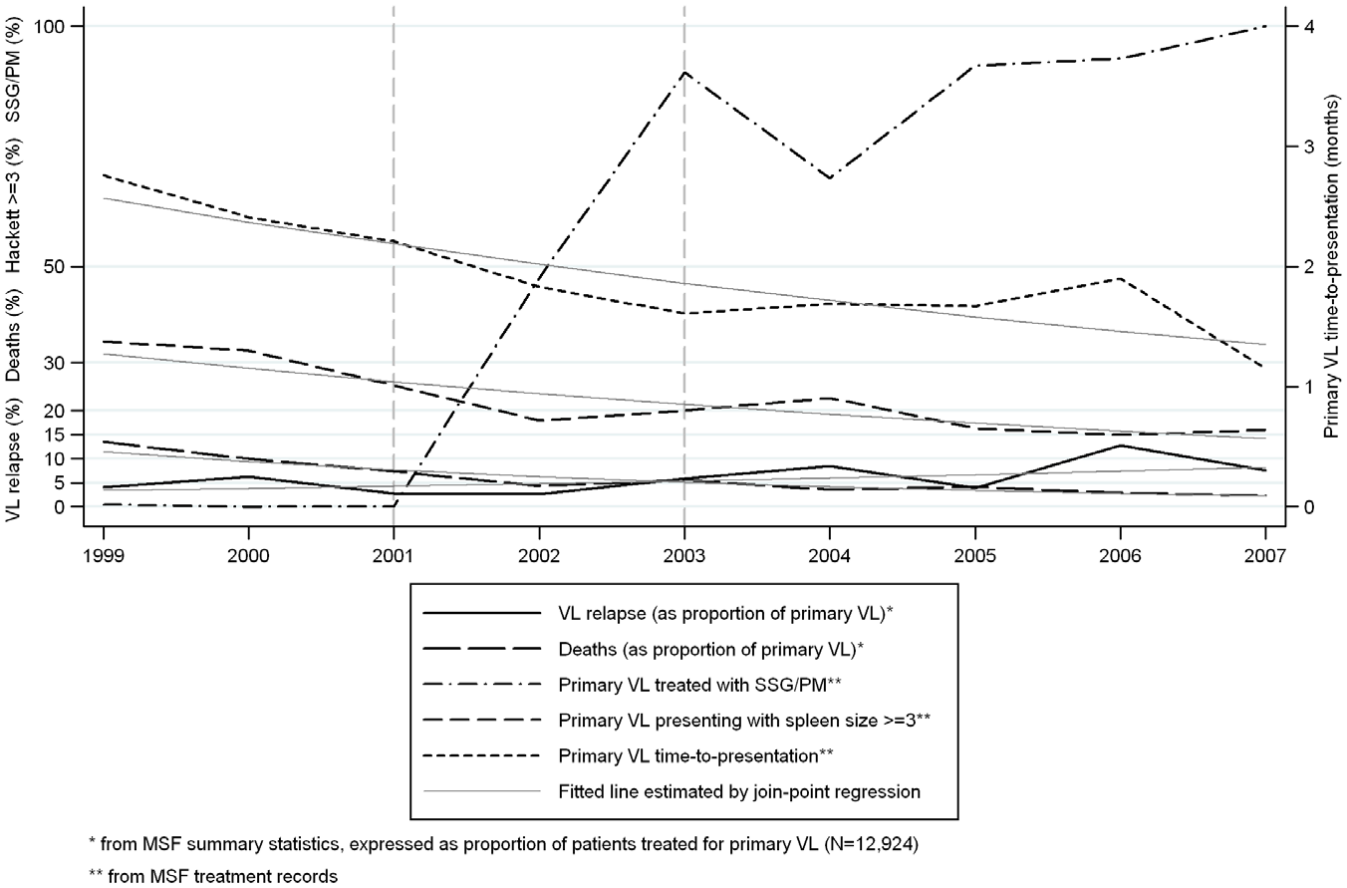
Trends in VL relapse and related factors in Southern Sudan (1999–2007).

**Table 6** shows summary statistics for the proportion of patients admitted to MSF treatment centres in Southern Sudan who were diagnosed with VL relapse (expressed as a proportion of patients treated for primary VL). Join-point analysis provided weak evidence (P = 0.12) for an upward trend in this proportion (annual percent change (APC) = 11.4% (−3.4% to 28.5%)), as illustrated in **Figure 2**. The proportion of patients who died fell dramatically between 1999 and 2001 (from 13.5% to 7.4%) and then declined steadily to <3% in 2006/2007. Join-point analysis provided strong evidence (P<0.001) for a downward trend in deaths during treatment for VL over the period 1999–2007 (APC = −18.1% (−22.5% to −13.4%), P<0.001) (**Table 6** **,** **Figure 2**). These trends in relapse and deaths were corroborated by logistic regression models (including age, sex, and treatment centre), which showed no discernible trend in risk of relapse (OR = 1.04 (0.95–1.14), P = 0.4), but a clear decline in risk of death (OR = 0.81 (0.77–0.86) per year, P<0.001).

**Table 6:** Trends in primary and relapse VL in Southern Sudan (1999–2007).

		1999	2000	2001	2002	2003	2004	2005	2006	2007	APC^h^
**Relapse VL** a		4.1%	6.3%	2.8%	2.6%	5.9%	8.5%	4.0%	12.8%	7.6%	11.4% (−3.4% to 28.5%)
**SSG** b		99.5%	100.0%	99.9%	52.3%	9.6%	31.6%	8.2%	6.7%	0.0%	-
**SSG/PM** b		0.5%	0.0%	0.1%	47.7%	90.4%	68.4%	91.8%	93.3%	100.0%	-
**Deaths** c		13.5%	10.0%	7.4%	4.4%	5.6%	3.6%	4.2%	2.9%	2.4%	18.1% (−22.5% to −13.4%)
**Mean time-to-presentation of VL relapse patients (months)** d		3.0	3.3	2.2	2.7	1.8	2.1	1.8	2.4	1.6	−6.7% (−11.3% to −1.8%)
**Mean time-to-presentation of primary VL patients (months)** e		2.8	2.4	2.2	1.8	1.6	1.7	1.7	1.9	1.2	−7.7% (−3.8% to −11.6%)
**Hackett Grade ≥3** f		34.4%	32.5%	25.3%	18.0%	20.0%	22.6%	16.4%	15.0%	16.0%	−9.6% (−13.5% to −5.5%)
**Interval between primary and relapse VL (days)** g	**median**	111	86	109	104	101	77	78	78	48	-
	**(range)**	(33–198)	(24–183)	(23–250)	(34–623)	(19–1358)	(27–145)	(45–310)	(22–240)	(48)	
	**patients**	n = 13	n = 4	n = 18	n = 40	n = 51	n = 13	n = 19	n = 7	n = 1	

a from MSF summary statistics (expressed as proportion of patients treated for primary VL, N = 11,319).

b from MSF treatment records (proportion of primary VL patients receiving this treatment, N = 8,521).

c from MSF summary statistics (expressed as proportion of all patients treated, N = 12,924).

d from MSF treatment records (duration of illness self-reported by VL relapse patients, N = 589).

e from MSF treatment records (duration of illness self-reported by primary VL patients, N = 7,841).

f from MSF treatment records (spleen size measured in primary VL patients, N = 8,494).

g from MSF treatment records (interval between discharge and re-admission, N = 166).

h Annual Percentage Change (95% confidence interval) estimated from join-point regression.

The proportion of primary VL patients presenting with splenomegaly indicated by Hackett Grade ≥3 decreased from 34.4% in 1999 to 16.0% in 2007 (**Table 6** **,** **Figure 2**); a trend of −9.6% (−13.5% to −5.5%) per annum (P<0.001). The mean time-to-presentation for primary VL patients fell from 2.8 to 1.2 months, and for relapse patients from 3.0 months in 1999 to 1.6 months in 2007 (**Table 6**). Join-point analysis showed a steady downward trend in the mean time-to-presentation of VL relapse patients (APC = −6.7% (−11.3% to −1.8%), P = 0.003) and of primary VL patients (APC = −7.7% (−3.8% to −11.6%), P = 0.003) (**Table 6** **,** **Figure 2**). **Figure 2** also shows that the proportion of primary VL patients treated with SSG/PM combination therapy, instead of SSG monotherapy, increased from <1% in 2001 to 90% in 2003 (**Table 6**). We found no trends in age and sex or in nutritional and walking status.

The median interval between discharge from treatment for primary VL and re-admission for VL relapse was 91 days (range 19–1358 days, N = 166) (**Table 6**); the median interval in years 1999–2003 (105 days) was longer (by 27 days) than in years 2004–2007 (Kruskal-Wallis test, 1df, P = 0.04). The overall proportion of patients relapsing within 3 months was 49.4%; within 6 months 83.7%; and within 12 months 96.4%. The interval between discharge from treatment for primary VL and re-admission for relapse was not associated with age, sex, spleen size on discharge, nutritional status on discharge or drug regimen.

## Discussion

We have shown that splenomegaly among patients with primary *L. donovani* VL was strongly associated with a much higher relative risk of VL relapse. Primary VL patients presenting with splenomegaly of Hackett Grade ≥3 had almost four-fold higher odds of subsequent relapse than patients with no enlargement of the spleen, and patients discharged with greatly enlarged spleens had greater than five-fold higher odds of relapse. The latter, but not the former, association remained evident after excluding from our analysis all patients who had no palpable spleen on admission (**Table 5**). No other clinical characteristics of primary VL patients emerged as strong risk factors for VL relapse, although infants appeared to be more susceptible as reported for *L. chagasi* VL [19].

Splenomegaly on admission may indicate a combination of severity of illness, parasite burden, and severity of immunosuppression. Splenomegaly on discharge suggests the patient has not responded adequately to treatment, and may either harbour a significant parasite burden or may still be immunosuppressed by the disease. Splenomegaly is one of the classical signs of VL, reported globally as present in >90% of VL patients [13]. Extremely enlarged spleen on admission is a sign of advanced disease and is a risk factor for death [3]. The spleen in *L. donovani* infection is infiltrated by parasitized macrophages as well as plasma cells, immune complexes and other components of immune response, leading to hyperplasia of reticulo-endothelial cells and enlargement of the organ [20]. A chronic inflammatory state mediated mainly by TNF results in architectural damage and immunological dysfunction [21], [22]. Particularly important for treatment outcome is loss of spleen marginal zone macrophages, which play an important role in capturing blood-borne pathogens; during enlargement, the spleen's protective role is reduced [23], [24]. Through clinical and pathophysiological observation, reduction of spleen size is recognised as one of the most important signs of successful treatment, and splenectomy has traditionally been practiced in splenomegalic patients who have frequent relapses despite appropriate treatment [25]. However, according to the WHO Manual on Visceral Leishmaniasis, a completely unpalpable spleen is not considered necessary to classify a patient as cured, (“persistently enlarged spleen is no cause for concern provided the patient's other symptoms are improving”) [13].

Severe anaemia is associated with the increased risk of death among VL patients [3] and, apart from other reasons (bone marrow suppression, malnutrition and generalised inflammation), is related to intensive destruction of erythrocytes in enlarged spleens [24]. Thus, the correlation between marked splenomegaly and anaemia found in our data would be expected. However, other indicators of advanced disease on admission, such as severe malnutrition and general weakness expressed as walking status, were not associated with relapse in our patients. Weight gain is a sign of clinical response to treatment, and mean percentage weight gain was marginally lower in the group of patients who experienced relapse. However, we note that discharge weight data were missing for 50% of patients. The mean percentage gain in haemoglobin level was the same in both groups, although data were missing for 80% of patients. Other signs of clinical response, such as improvement of general status and becoming afebrile, were not recorded in our database.

We found that 17-day SSG/PM combination therapy was associated with two-fold higher odds of relapse than 30-day SSG monotherapy. The joinpoint analysis showed an upward trend in VL relapse admissions as a proportion of primary VL admissions (**Figure 2**), which might be interpreted as a consequence of the introduction of SSG/PM as first-line treatment for primary VL between 2001 and 2003. However, this apparent trend was driven by the relatively high proportion of VL relapse patients treated in the last two years (2006–2007), and the risk factor analyses showed no discernible trend. The 17-day SSG/PM regimen was introduced on the basis of evidence from several trials [26]–[28], and in a retrospective study (2002–2005) based on MSF treatment data from Southern Sudan, the SSG/PM regimen was found to give better survival and cure rates than SSG alone [9]. Preliminary analysis of a phase III clinical trial by the Drugs for Neglected Diseases Initiative (DNDi) indicated as yet no difference in cure rates between 17 days of SSG/PM and 30 days of SSG [DNDi - unpublished data]; final results of the trial are due in 2010 [29]. It could be that the very patients who were saved from dying by SSG/PM may be those who relapsed. This phenomenon probably occurred during our study of miltefosine *vs* SSG in Ethiopia: miltefosine, being a safer drug than SSG, was associated with a far lower death rate, yet a far higher relapse rate [30]. However, not all previously-reported risk factors for death were evident as risk factors for relapse (possibly because drug toxicity is a primary cause of death) [3], and we cannot discount a possible role of shorter duration of treatment in increasing risk of relapse [31].

Our reported association between SSG/PM and risk of VL relapse was likely to be residually confounded, and possibly biased by missing data, because SSG/PM was used at permanent MSF treatment centres, whereas SSG monotherapy was used at temporary (seasonal) outreach sites. Access to care in the event of VL relapse was much easier at the permanent sites, some of which have even benefited from public transport since 2005. Temporary sites were also less able to diagnose relapses, because the splenic or lymph node aspirates which are necessary to make the diagnosis of relapse could only be carried out at the main treatment centres. These factors, together with better record-keeping, probably explain the apparently higher risk of relapse associated with treatment at Lankien.

The use of SSG/PM during the latter half of the period in our study coincided with a series of political agreements (between 2003 and 2005), which brought a tentative ceasefire to Southern Sudan after decades of conflict. Hence, the crude upward trend seen in MSF's summary data is probably a consequence of easier access to treatment, as reflected in the trend towards shorter ‘time-to-presentation’ for VL patients (**Figure 2**). In the past, when access to care was restricted, patients with relapse may have died without reaching a treatment centre. HIV infection was unlikely to be a factor in VL relapse during most of the period in our study. An unlinked screening study of 206 VL patients in Lankien in 2002 revealed only 1 HIV-positive case (0.5%); or 1 in 36 adult patients (2.8%) [MSF - unpublished data]. However, the number of VL patients has declined in recent years (from 4,172 in 2003 to 101 in 2008), while large numbers of refugees have begun to return to Southern Sudan from places with higher HIV prevalence (Ethiopia, Kenya, Khartoum). Hence we might expect the proportion of VL relapses attributable to HIV co-infection to increase. Recent (2008) data from Nasir, where MSF began routine HIV testing and anti-retroviral therapy in adult VL patients, showed that 25% (5/20) were HIV co-infected [MSF - unpublished data].

Our study was the largest retrospective analysis of VL relapse globally. The difficult conditions under which the data were collected gave rise to some limitations, principally missing treatment records, missing data and, for this study, our inability to link all VL relapse patients with their record of treatment for primary VL. This was mainly because the majority of patients did not present the identification card given to them when they were discharged from treatment for primary VL. It is difficult to assess the impact of missing records and missing data on our outcomes, although we have no reason to suspect these were a source of significant bias in our analysis of clinical risk factors. Some of the primary VL patients whom we classified as “primary VL patients who did not subsequently relapse” may have had an untreated or unrecorded relapse, and some patients who had been previously treated may have been diagnosed as primary VL if the patient did not recall their primary episode. These two misclassifications would lead to under-estimation of risk factors for relapse. The 5 patients (3%) re-admitted soon after discharge (19 to 31 days) may have been treatment failures, rather than relapses. Through our sensitivity analysis we attempted to adjust for those patients with no palpable spleen, who were possibly falsely diagnosed as VL patients (hence at zero risk of relapse). However, in a setting with high prevalence of other endemic diseases which cause splenomegaly (malaria, typhoid, schistosomiasis, brucellosis, and liver cirrhosis), some patients with splenomegaly may also not have had primary VL. We could not conduct a further sensitivity analysis, based on the actual diagnostic method for each patient (parasitological, immunological, clinical), because this could not be deduced from the available data. We noted that relapse VL patients for whom we could not identify a previous treatment record had less severe splenomegaly on re-admission than relapse VL patients whose previous treatment data were used in our analysis. However, spleen size on admission for relapse VL was not correlated with spleen size at end of treatment for primary VL, hence this difference would not have led to an over-estimate of the association between splenomegaly at end of treatment for primary VL and subsequent relapse.

Our finding that SSG/PM is associated with increased risk of relapse is likely to be confounded by improvements in access to treatment which coincided with the introduction of this shorter combination drug regimen, and may reflect better survival rates compared with SSG monotherapy. We await a definitive answer from a randomised controlled trial of SSG/PM *vs* SSG currently underway in East Africa. Meanwhile, our finding that splenomegaly is associated with increased risk of VL relapse could contribute to revised guidelines for clinical assessment of VL patients prior to discharge.
